# Supplementary material for: MetaRibo-Seq measures translation in microbiomes
Source: Nat Commun. 2020 Jun 29;11:3268. doi: 10.1038/s41467-020-17081-z (PMC7324362; doi:10.1038/s41467-020-17081-z)
Supplement: Supplementary file 10 — Supplementary Data 7 [file 41467_2020_17081_MOESM10_ESM.zip › File2/Confidence_High_Taxonomy/443474_out.krona.html]

Javascript must be enabled to view this page.

members
magnitude
magnitudeUnassigned
count
unassigned
taxon
rank

443474\_out

8

2
8
superkingdom

8
1239
phylum

186801
8
class

186802
8
order

186803
8
family

genus
1407607
2

1150298
2

SRS077454\_contig\_number\_1944SRS148159\_contig\_number\_49936
species

6
572511
1
genus

SRS097920\_contig\_number\_7069


SRS1055043\_contig\_number\_contig-100\_3675.141377
1
1896976
species

species
2292986
1

SRS017103\_contig\_number\_4159

species

SRS104400\_contig\_number\_39393
1
2292966

species
1
2292971

SRS1041036\_contig\_number\_1094


SRS062654\_contig\_number\_26458
1
2292958
species
